# Clinical features, complications, and outcomes of exogenous and endogenous catecholamine‐triggered Takotsubo syndrome: A systematic review and meta‐analysis of 156 published cases

**DOI:** 10.1002/clc.23352

**Published:** 2020-03-03

**Authors:** Shams Y‐Hassan, Henrik Falhammar

**Affiliations:** ^1^ Coronary Artery Disease Area, Heart and Vascular Theme Karolinska Institutet and Karolinska University Hospital Stockholm Sweden; ^2^ Department of Endocrinology, Metabolism and Diabetes Karolinska University Hospital Stockholm Sweden; ^3^ Departement of Molecular Medicine and Surgery Karolinska Institutet Stockholm Sweden

**Keywords:** broken heart syndrome, epinephrine, myocardial stunning, norepinephrine, paraganglioma, pheochromocytoma, Takotsubo

## Abstract

Innumerable physical stress factors including externally administered catecholamines, and pheochromocytomas and paragangliomas (PPGLs) have been reported to trigger Takotsubo syndrome (TS). A systematic search of PubMed/MEDLINE identified 156 patients with catecholamine‐induced TS up to December 2017. Data were compared within the catecholamine‐induced TS cohort, but some comparisons were also done to a previously published large all‐TS cohort (n = 1750). The mean age was 46.4 ± 16.4 years (72.3% women). The clinical presentation was dramatic with high complication rates in (68.2%, n = 103; multiple complications 34.6%, n = 54). The most common TS ballooning pattern was apical or mid‐apical (45.2%, n = 69), followed by basal pattern (28.8%, n = 45), global pattern (16.0%, n = 25), mid‐ventricular (8.3%, n = 13), focal (0.6%, n = 1), and unidentified pattern (1.9%, n = 3). There was an increase in the prevalence of apical sparing ballooning pattern compared to all‐TS population (37.7% vs 18.3%, *P* < .00001). Higher complication rates were observed in TS with global ballooning pattern compared to apical ballooning pattern (23/25, 92% vs 38/65, 58.5%; *P* = .0022). Higher complication rates were observed in patients with age < 50 years than patients >50 years (73/92, 79.3% vs 29/56, 51.8%, *P* = 0.0009). Recurrence occurred exclusively in patients with PPGL‐induced TS (18/107 patients, 16.8%). PPGL‐induced TS was characterized by more global ballooning's pattern (22/104, 21.2% vs 3/49, 6.1%, *P* = 0.02), and lower left ventricular ejection fraction (25.54 ± 11.3 vs 31.82 ± 9.93, *P* = 0.0072) compared to exogenou**s** catecholamine‐induced TS. In conclusion, **c**atecholamine‐induced TS was characterized by a dramatic clinical presentation with extensive left ventricular dysfunction, and high complication rate.

## 1 BACKGROUND

Takotsubo syndrome (TS) is an acute cardiac disease entity with a clinical presentation resembling that of an acute coronary syndrome.^1^, ^2^ The syndrome is characterized by a striking regional left ventricular wall motion abnormality (LVWMA) with a circumferential pattern extending beyond the coronary artery supply territory and resulting in a conspicuous ballooning of the left ventricle during systole.^3^, ^4^ Innumerable physical stress factors have been reported to trigger the disease.^5^ Among the physical stressors are external administration of epinephrine^6^ and norepinephrine^7^ and the disease conditions causing increased catecholamine elevations, sometimes massively, such as pheochromocytomas and paragangliomas (PPGLs).^8^, ^9^, ^10^

PPGLs are catecholamine‐secreting tumors that arise from chromaffin tissue of the sympathetic nervous system.^11^, ^12^ PPGLs presentation may be vague and the interpretation of the symptoms and signs may be difficult.^13^ The classic triad (hypertension, hyperhidrosis, and palpitation) and paroxysmal hypertension were the symptoms that usually lead to the suspicion of PPGLs previously.^13^ PPGLs are more frequent in certain groups, for example, in patients with adrenal incidentalomas with 0.6% to 4.2% being affected but is otherwise generally rare.^14^, ^15^, ^16^ Most PPGLs are nowadays diagnosed due to an incidentaloma, then due to catecholamine excess symptoms and finally because of screening in a previously known familial syndrome (eg, multiple endocrine neoplasia type 2, von Hippel Lindau syndrome, neurofibromatosis type 1, and mutations in succinate dehydrogenase B, C, and D)^12^, ^13^, ^17^, ^18^, ^19^ Cushing's syndrome due to ectopic ACTH‐production from a PPGL can occasionally occur,^20^, ^21^ and thus all adrenal tumors should have a 1 mg overnight dexamethasone suppression test to exclude cortisol excess.^22^, ^23^ Sometimes an adrenal medullary hyperplasia may be the culprit of catecholamine excess.^24^

The data on catecholamine‐induced TS are limited and mostly consist of case reports and case series. Thus, in this systematic review and meta‐analysis, the clinical features, complications and outcomes of 156 published cases of externally administered epinephrine‐ and norepinephrine‐, and PPGL‐induced TS are described. Furthermore, a comparison was made between the whole group and a study including all‐TS population. The externally administered catecholamine‐induced TS is also compared to the PPGL‐induced TS.

## 2 METHODS

**Figure 1:**
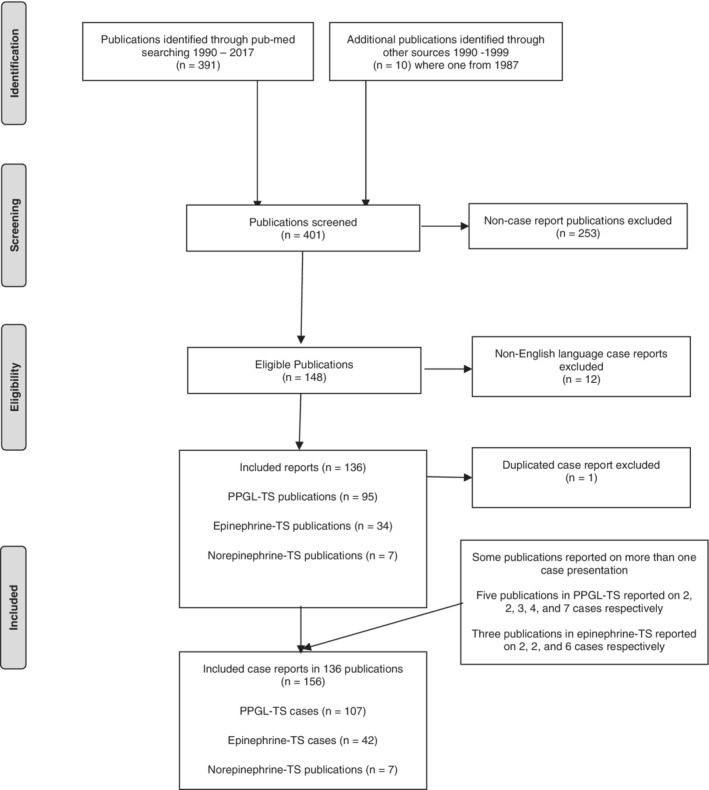
Flow chart illustrating the procedure for article inclusion and exclusion in a systematic review of cases of pheochromocytoma and paraganglioma (PPGL)‐, epinephrine‐, and norepinephrine‐triggered takotsubo syndrome (TS)

All cases of epinephrine‐induced TS, norepinephrine‐induced TS, and PPGL‐induced TS from 1990, the year where the Japanese term Takotsubo was introduced, to December 2017 were critically reviewed. The cases were retrieved by systematical searches in PubMed/Medline using the search terms “Takotsubo,” “apical ballooning,” “stress cardiomyopathy,” and “broken heart syndrome,” and linking them with the terms “pheochromocytoma,” “paraganglioma,” “catecholamines,” “epinephrine,” “adrenaline,” “norepinephrine,” and “noradrenaline.” Cases with PPGL‐induced transient left ventricular dysfunction where the clinical features and course were consistent with TS were also included. Articles were initially screened by title for relevance and then by abstract, with full‐text articles of potentially relevant reports reviewed. The reference lists of the retrieved full‐text studies were scanned to identify additional relevant reports. Only case‐reports or reports on a series of cases where enough information was available on every case were included (Figure 1).

The following information was extracted from the publications: the age and gender of the patients, the clinical presentation, the type of ECG changes and the cardiac biomarkers in all patients. The TS localization was deemed by the description in the text or the available figures in the manuscripts. The results were compared with 1750 patients from the study by Templin et al^5^ where all types of TS (all‐TS) were included, when comparable information was available in both studies. Furthermore, the 49 cases with externally administered epinephrine‐ or norepinephrine‐induced TS were compared with the 107 cases of PPGL‐induced TS. Quality assessment tools such as the Newcastle‐Ottawa Scale (available at: http://www.ohri.ca/programs/clinical_epidemiology/oxford.asp) was considered inappropriate because these instruments had not been developed to study case reports or series. The PRISMA (Preferred Reporting Items for Systematic Reviews and Meta‐Analyses) guidelines were followed.

### 2.1 Statistical analysis

Continuous variables are presented as means ± standard deviations and categorical data as absolute values and percentages. Fisher's exac*t* test or Chi‐square test was used as appropriate to compare categorical data, and two‐tailed unpaired student's *t* test was used for continuous variables. A *P* < .05 was considered significant.

## 3 RESULTS

The systematic search identified 391 potentially relevant records, with an additional 10 records (including one case of PPGL‐induced TS from 1987^25^ with features typical for mid‐apical TS) identified through review of the reference lists. After excluding all report not reporting on original cases, 148 articles were screened for eligibility. Further 12 non‐English case reports were then excluded since information could not be extracted. Consequently, 136 publications were included; 8 publications reported on more than one case report (from 2 to 7 cases; Figure 1). Because of the extreme similarities between two reported pheochromocytoma‐induced TS cases,^26^, ^27^ one case which was reported later was excluded.^27^ In total, 156 cases reports (107 cases with PPGL‐induced TS,^9^, ^28^, ^29^, ^30^, ^31^, ^32^, ^33^, ^34^, ^35^, ^36^, ^37^, ^38^, ^39^, ^40^, ^41^, ^42^, ^43^ 42 cases epinephrine‐induced TS,^6^, ^44^, ^45^, ^46^, ^47^, ^48^, ^49^, ^50^, ^51^, ^52^ and 7 cases norepinephrine‐induced TS^53^, ^54^, ^55^, ^56^, ^57^, ^58^, ^59^) constitute the patient cohort for the meta‐analysis. Among the cases included were two non‐English case reports (one case in Swedish^60^ and one in German^61^) where enough information could be obtained. Ten of 156 of the cases (9 PPGLs and 1 epinephrine), were deemed to be catecholamine‐induced TS but were reported before the TS‐era, that is, cases reported before 1999 when the first reports on TS were published in English. The remaining 146 cases were reported during the TS‐era.

### 3.1 Clinical picture and presentation

The mean age was 46.4 ± 16.4 years (range 16‐86 years). Women constituted 72.3% of the cohort. In all the patients, either external epinephrine (n = 42) or norepinephrine (n = 7) administration or PPGLs (n = 107) was documented as the most probable physical trigger factor for TS. However, in 75 of 156 patients (48.1%) additional potential trigger factors were identified (emotional stressors in 8 cases [all PPGLs] and physical stressors in 67 cases). In most of the patients with external epinephrine‐ or norepinephrine‐induced TS, the disease conditions, which indicated catecholamine administration, could have been a potential trigger factor for TS.

The most common clinical presentation was chest pain, which occurred alone in 22.4% (n = 35) of the patients. Other presenting symptoms were syncope (6.4%, n = 10), dyspnea in (4.5%, n = 7), abdominal pain (3.8%, n = 6), and headache (3.8%, n = 6). Signs and symptoms suggestive of PPGLs (such as dizziness, palpitation, profuse sweating, pallor, headache and hypertension)^13^ alone were the presenting symptom in 6.4% (n = 10; exclusively PPGL patients). Signs and symptoms suggestive of PPGLs were associated with chest pain in further 19.2% (n = 30), with abdominal pain in 5.1% (n = 8), and with dyspnea in 4.5% (n = 7). Severe hemodynamic compromise was the presenting feature in 6.4% (n = 10; pulmonary edema 2.6% n = 4, circulatory failure 1.9% n = 3, and cardiogenic shock 1.9% n = 3). Arrhythmias, including ventricular tachycardia, occurred in 4.5% (n = 7), and other ECG changes were the presenting features in another 2.6% (n = 4). Other presenting symptoms were cough (1.9%, n = 3), hypoxia (1.3%, n = 2), and fever (0.6%, n = 1).

Information on electrocardiographic (ECG) changes was available in 141 patients (90.4%; Table 1). Among important ECG changes were ST‐elevation myocardial infarction (STEMI)‐like changes (35.5%, n = 50), T‐wave inversion (17.7%, n = 25), ST depression (22.7%, n = 32), peaked T waves (3.5%, n = 5), and sinus tachycardia (7%, n = 10). Non‐specific ECG changes was found in 7% (n = 10). Normal ECG was found in only 3.5% (n = 5). The most common ballooning pattern was apical or mid‐apical (45.2%, n = 69) followed by basal (inverted) pattern (28.8%, n = 45), global pattern (16.0%, n = 25), mid‐ventricular (8.3%, n = 13), focal (0.6%, n = 1), and unidentified pattern (1.9%, n = 3). The left ventricular ejection fraction (EF), which was available in 105 patients, was markedly reduced to 27.51 ± 11.23%.

**Table 1:** Catecholamine‐induced takotsubo syndrome (TS) compared with all‐TS (Templin et al^5^), and pheochromocytoma/paraganglioma‐induced TS compared to combined epinephrine/norepinephrine drug‐induced TS

	PPGL‐E/NE‐induced TS	All‐TS (Templin et al,^5^)	*P* values^a^	PPGL‐TS	Combined E/NE‐TS	*P* value^b^
	(n = 156)	(n = 1750)		(n = 107)	(n = 49)	
*Mean age (y)*	46.37 ± 16.40	66.4 ± 13.1	<.0001	47.1 ± 15.9	44.81 ± 17.53	.42
	(16–86)			(16‐86)	(16‐81)	
	(n = 153)			(n = 107)	(n = 49)	
*Gender*, *female (n)*	112 (72.3%)	1571 (89.8%)	<.00001	78 (72.9%)	34 (70.8%)	.85
	(n = 155)				(n = 48)	
*Presenting symptom*						
*chest pain (n)*	65 (41.7%)	1226 (75.9%)	<.00001	46 (43.0%)	19 (39.6%)	.73
		(n = 1619)			(n = 48)	
Heart rate (beats/min)	114.0 ± 29.8	87.5 ± 21.8	<.0001	117.7 ± 30.1	104.7 ± 27.4	.0759
	(n = 80)			(n = 57)	(n = 23)	
*Precipitating stressors*						
Emotional (n)	c	485 (27.7%)	N/A	c	c	
Physical (n)	156 (100%)	630 (36.0%)	N/A	107 (100%)	49 (100%)	NS
Both emotional and physical (n)		137 (7.8%)	N/A	N/A	N/A	
No triggers (n)	0 (0%)	498 (28.5%)	N/A	0 (0%)	0 (0%)	NS
*ECG changes*						
STEMI‐like changes (n)	50 (35.5%)	690 (43.7%)	.06	35 (36.5%)	15 (33.3%)	.85
	(n = 141)	(n = 1578)		(n = 96)	(n = 45)	
ST‐depression (n)	32 (22.7%)	131 (8.3%)	<.00001	25 (26,0%)	7 (15,6%)	.20
	(n = 141)	(n = 1578)		(n = 96)	(n = 45)	
*Myocardial infarction biomarkers (n)*	126 (97.7%) (n = 129)	87.0%	.0001	83 (96.5%) (n = 96)	43 (100%) (n = 45; N/A n = 6)	.55
*Ejection fraction (%)*	27.5 ± 11.2 (n = 105)	41.1% ± 11.8	<.0001	25.5 ± 11.3 (n = 72)	31.8 ± 9.9 (n = 33)	.0072
*TS localization pattern (n)*	(n = 153)	(n = 1750)		(n = 104)	(n = 49)	
Apical (n)	69 (45.2%)	1430 (81.7%)	<.00001	47 (43.9%)	22 (44.9%)	.90
Mid‐ventricular (n)	13 (8.3%)	255 (14.6%)	.039	6 (5.6%)	7 (14.3%)	.12
Basal (n)	45 (28.8)	39 (2.2%)	<.00001	28 (26.2%)	17 (34.7%)	.35
Global (n)	25 (16.0%)	0 (0%)	<.00001	22 (20.6%)	3 (6.1%)	.02
Focal (n)	1 (0.6%)	26 (1.5%)	.72	1 (0.9%)	0 (0%)	1.0
Unidentified (n)	3 (1.9%)			3 (2.8)		
*Combined in‐hospital complications (n)*	103 (68.2%) (n = 151)	374(21.8%) (1716)	<.00001	74 (71.8%) (n = 103)	29 (60.4%) (n = 48)	.19
*Cardiogenic shock (n)*	57 (37.7%) (n = 151)	170 (9.9%) (n = 1716)	<.00001	41 (39.8%) (n = 103)	16 (33.3%) (n = 48)	.48
*Death (n)*	6 (3.8%) (n = 156)	72 (4.1%) (n = 1750)	1.0	4 (3.7%) (n = 107)	2 (4.1%) (n = 49)	1.0
*Inotropic medications (n)*	51 (32.7%) (n = 156)	212 (12.2%) (n = 1735)	<.00001	35 (33.7%) (n = 104)	15 (30.6%) (n = 49)	.85
*TS recurrence rate (n)*	19 (12.3%) (n = 155)	57 (3.3%)	<.00001	18 (16.8%)	1^d^	N/A

Abbreviations: ECG, electrocardiogram; NA, not available; N/A, not applicable; PPGL‐E/NE, Pheochromocytoma and paraganglioma‐Epinephrine/Norepinephrine; STEMI, ST‐elevation myocardial infarction; TS, takotsubo syndrome.

a *P* value PPGL‐E/NE‐TS vs all‐TS.

b *P* value PPGL‐TS vs Combined E/NE‐TS.

c Please see the text regarding other potential trigger factors.

d May be recurrent or may be induced by norepinephrine.

STEMI‐like ECG changes and T‐wave inversions were significantly more prevalent in apical ballooning pattern compared to basal ballooning pattern (STEMI n = 34 and T‐wave inversions n = 19 in 69 patients with apical ballooning vs STEMI n = 3 and T‐wave inversion n = 1 in 45 patients with basal ballooning pattern, *P* < .00001). In contrast, ST‐depression and peaked T waves were significantly more prevalent in basal ballooning pattern compared to that of apical ballooning's pattern (ST‐depression n = 23 and peaked T‐waves n = 5 in 45 patients with basal ballooning pattern vs ST‐depression n = 4 and peaked T‐waves n = 0 in 69 patients with apical ballooning pattern, *P* < .00001).

The myocardial “infarction” biomarkers were in available in 129 patients (82.7%). It was increased in 97.7% (n = 126). In 121 patients (77.5%), information on coronary angiography was available. In 98.3% (n = 119), the coronary angiography was normal. Only 1.7% (n = 2) had signs of obstructive coronary artery disease.

### 3.2 Complications and outcomes

Information on complications was available in 151 of 156 patients (96.8%). Complications occurred in 68.2% (n = 103) (Table 1). Multiple complications were found in 34.6% (n = 54); the most common combination of complications were heart failure, pulmonary edema, cardiogenic shock, and circulatory and respiratory failure. Heart failure occurred in 53.0% (n = 80), cardiogenic shock in 37.7% (n = 57), pulmonary edema in 37.1% (n = 56), respiratory failure in 12.6% (n = 19), thrombo‐embolic complications in 9.3% (n = 14), arrhythmias including ventricular tachycardia in 8.6% (n = 13), cardiac arrest in 6.6% (n = 10), metabolic acidosis in 4.6% (n = 7), multiple organ failure in 4.0% (n = 6), electro‐mechanic dissociation in 2.6% (n = 4), and left ventricular outlet tract obstruction in 0.7% (n = 1). Significantly higher complication rates were observed in TS with global ballooning pattern compared to apical ballooning pattern (23/25, 92.0% vs 38/65, 58.5%; *P* = .0022). The complications occurred in 71.1% (32/45) of the basal ballooning pattern of TS compared to 58.5% (38/65) of the apical ballooning pattern (*P* = .227). There was no difference in the rate of complications between men and women (69.8% vs 67.3%; *P* = .85). In contrast, the complication rates were higher in patients aged <50 years than patients aged >50 years (73/92, 79.3% vs 29/56, 51.8%; *P* = .0009). Inotropic medications were used in 33.1% (50/151). The most common used inotropic medication alone or in combinations were dobutamine, norepinephrine, epinephrine, amrinone, or milrinone. Mechanical ventilation was used in 25.8% (34/132). Extracorporeal life support (ECLS), veno‐arterial extra‐corporeal membrane oxygenation (ECMO), percutaneous cardiopulmonary support, left ventricular assist device, and intra‐aortic balloon pump were used alone or in combination in 21.5% (31/144). Recurrence occurred exclusively in patients with PPGL‐induced TS (16.8%, 18/107).

In total 3.8% (n = 6) died; 7% in the male group (n = 3), and 2.7% (n = 3) in the female group (*P* = .35). Death occurred in 3.7% (n = 4) of the patients with PPGL‐, 4% (n = 2) with exogenous catecholamine‐induced TS. Mortality during recurrence of PPGL‐induced TS was high (11%, 2/18). Different inotropic medications alone or in combinations were used in most patients who died (67%, 4/6); this may have contributed to the deaths.

### 3.3 Catecholamine‐induced TS vs all‐TS population

Compared to all‐TS population,^5^ the patients in catecholamine‐induced TS were 20 years younger (*P* < .0001; Table 1). The TS prevalence in men was increased to 27.7% in catecholamine‐induced TS compared to 10.2% in all‐TS population; however, women were still predominating. The disease was more severe in catecholamine‐induced TS with significantly higher heart rate and lower left ventricular ejection fraction; 16.3% of patients had global left ventricular dysfunction compared to 0% in all‐TS population.^5^ There was also increase in the prevalence of apical sparing ballooning pattern compared to all‐TS population (37.7% vs 18.3%, *P* < .00001). The disease severity was reflected in significantly higher complication rate (68.2% vs 21.8%), more cardiogenic shock (37.7% vs 9.9%) and higher use of inotropic medications (32.7% vs 12.2%). Significantly higher recurrence rate was reported in catecholamine‐induced TS (all in patients with PPGL‐induced TS) than all‐TS population.

### 3.4 Exogenous catecholamine‐induced TS vs PPGL‐induced TS

The disease in PPGL‐induced TS was more severe with significantly increased prevalence of global TS‐pattern and lower left ventricular ejection fraction (Table 1). Complication rates were higher in PPGL‐induced TS, but this did not reach significant levels.

## 4 DISCUSSION

This systematic review and meta‐analysis reports hitherto on the largest number of patients (n = 156) with exogenous (epinephrine and norepinephrine) catecholamine‐ and endogenous (PPGLs) catecholamine‐induced TS. The main findings were that a substantial number of patients presented with severe hemodynamic compromise such as pulmonary edema, cardiogenic shock, circulatory failure, and arrhythmias. Considerable numbers of patients had global left ventricular dysfunction and marked depression of left ventricular ejection fraction. This was reflected in high complication rates during admission where two‐thirds of the patients suffered complications and one third had multiple complications. Worth mentioning is that the description of LVWMA was not always accurate especially in the cases described before the TS‐era. Five out of nine PPGL‐induced TS published before 2000 were described to have myocardial depression or severe left ventricular dysfunction with a clinical course consistent with TS.^62^, ^63^, ^64^, ^65^, ^66^ The other four patients had typical apical or basal (inverted) TS pattern.^25^, ^67^, ^68^, ^69^ All cases with severe left ventricular dysfunction were deemed to have global TS in this study. Global TS induced by other physical factors has also been reported.^70^, ^71^ Patients with PPGL‐induced TS may deteriorate rapidly and the TS localization may transform from regional to global.^10^ Such change has been well‐demonstrated in a case where the patient had mid‐basal TS during the first admission day and this progressed rapidly to severe biventricular failure during the following day.^72^ Several cases with PPGL‐induced TS with such startling course complicated by respiratory failure, metabolic acidosis and cardiogenic shock have been reported.^73^, ^74^ In one study constituted of 140 patients with PPGLs,^75^ 15 patients (10.7%) suffered “acute catecholamine cardiomyopathy”. Six out of 15 patients displayed classical mid‐apical or mid‐basal (inverted) TS. The remainder had severe extensive or global hypokinesia and a clinical picture of pulmonary edema. These findings may indicate that patients with PPGL‐triggered global biventricular failure may in fact have global TS.

Higher complication rates were observed in patients aged <50 years than those >50 years. Mortality was higher in men (7%) than women (2.6%). These findings are in line with what other investigators have reported in TS in general.^5^

There was an increased prevalence of apical sparing ballooning pattern (37.7%; basal pattern in 28.8%, midventricular ballooning pattern in 8.3%, and focal pattern in 0.6%). In PPGL‐induced TS, high recurrence rate (16.8%) was reported. The high recurrence rate of TS in PPGL‐induced TS population was most likely attributed to the delay in the PPGL diagnosis where episodes of massive catecholamine elevation had acted as a trigger factor.^76^

The more severe disease in PPGL‐induced TS than in exogenous catecholamine‐induced TS may be attributed to higher catecholamine surge in the former.^9^ Unfortunately, it was not possible to evaluate the degree of catecholamine elevations in PPGL‐induced TS in comparison to that of externally administered catecholamines. However, it has been reported that in external epinephrine‐induced TS, the administration of >1 mg epinephrine was associated with significantly higher complication rate than administration of ≤1 mg epinephrine (92% vs 42.9%).^6^

### 4.1 Implication of the findings in catecholamine‐induced TS on the pathogenesis of TS

One hypothesis in the pathogenesis of TS is epinephrine‐induced switch in signal trafficking in the apical left ventricular myocardium causing apical ballooning.^77^ In the current systematic review and meta‐analysis epinephrine was involved in triggering TS in 42 patients with external epinephrine administration and most of the 107 patients with PPGL‐triggered TS. Interestingly, 37.7% of catecholamine‐induced TS had apical sparing TS pattern, which was significantly higher than 18.3% of all‐TS population.^5^ This finding strongly challenges epinephrine‐induced switch in signal trafficking in the apical left ventricular myocardium. The higher prevalence of apical sparing TS pattern in epinephrine‐induced TS and PPGL‐induced TS have also been reported in other studies.^6^, ^8^, ^9^, ^78^ Furthermore, almost all TS studies have, apart from PPGL‐induced TS, shown either normal or mild to moderate plasma epinephrine elevation.^79^ Consequently, it is justified to conclude that there is no direct causal relation between epinephrine and TS but epinephrine may, as any other physical trigger factor, induce TS through sympathetic nervous system hyper‐activation including cardiac sympathetic nerve terminals with norepinephrine seethe and spill over.^1^, ^10^, ^80^

### 4.2 Limitations

The analysis of catecholamine‐induced TS was based on retrospective studies of case reports or series with the inherent limitations of all retrospective studies, in particularly that of ascertainment bias. The absolute levels of the cardiac biomarkers and catecholamine levels could not be utilized for the estimation of comparable mean values because of lack of standardization and uniformity across the case reports. The TS localization pattern was not always accurate, and this point has been discussed previously.^81^ The possibility of additional trigger factors, which could have triggered TS, especially in patients with exogenous catecholamine‐induced TS, cannot be ruled out.^82^, ^83^

## 5 CONCLUSIONS

The clinical features, complications and outcome of the hitherto largest number of patients with exogenous and endogenous catecholamine‐induced TS are described in this systematic review and meta‐analysis. Catecholamine‐induced TS was characterized by a dramatic clinical presentation with extensive left ventricular dysfunction, and high complication rates.

## CONFLICT OF INTEREST

The authors declare they have no conflict of interest.
